# Creation of an Integrated Clinical Trial Database and Data Sharing for Conducting New Research by the Japan Lung Cancer Society

**DOI:** 10.1016/j.jtocrr.2022.100317

**Published:** 2022-03-27

**Authors:** Yuichi Ozawa, Nobuyuki Yamamoto, Kouji Yamamoto, Kentaro Ito, Hirotsugu Kenmotsu, Hidetoshi Hayashi, Takehito Shukuya, Daichi Fujimoto, Shunichi Sugawara, Seiji Niho, Yuichiro Ohe, Hiroaki Okamoto, Kazuhiko Nakagawa, Katsuyuki Kiura, Ichiro Yoshino, Akihiko Gemma

**Affiliations:** a Internal Medicine III, Wakayama Medical University, Wakayama, Japan; b Department of Biostatistics, Yokohama City University Graduate School of Medicine, Kanagawa, Japan; c Respiratory Center, Matsusaka Municipal Hospital, Mie, Japan; d Division of Thoracic Oncology, Shizuoka Cancer Center, Shizuoka, Japan; e Department of Medical Oncology, Kindai University Faculty of Medicine, Osaka, Japan; f Department of Respiratory Medicine, Juntendo University Graduate School of Medicine, Tokyo, Japan; g Department of Pulmonary Medicine, Sendai Kousei Hospital, Miyagi, Japan; h Department of Pulmonary Medicine and Clinical Immunology, Dokkyo Medical University School of Medicine, Tochigi, Japan; i Department of Thoracic Oncology, National Cancer Center Hospital, Tokyo, Japan; j Department of Respiratory Medicine, Yokohama Municipal Citizen’s Hospital, Kanagawa, Japan; k Department of Allergy and Respiratory Medicine, Okayama University Hospital, Okayama, Japan; l Department of General Thoracic Surgery, Chiba University Graduate School of Medicine, Chiba, Japan; m Department of Pulmonary Medicine and Oncology, Graduate School of Medicine, Nippon Medical School, Tokyo, Japan

**Keywords:** Database, Locally advanced, Non–small cell lung cancer, Japan Lung Cancer Society

## Abstract

**Introduction:**

Although data accumulated in clinical trials have higher accuracy compared with real-world data and are irreplaceably valuable, most previous clinical trial data have been left unused.

**Methods:**

The Japan Lung Cancer Society (JLCS) asked six clinical trial groups that conducted randomized clinical trials on curative chemoradiation for locally advanced NSCLC to provide data. After obtaining consent from all six groups, data were collected from August 2019 to June 2021.

**Results:**

A total of eight trials, JCOG9812, JCOG0301, NJLCG0601, OLCSG0007, WJTOG0105, WJOG5008L, SPECTRA, and TORG1018, were included. More than 3000 data items were integrated into 408 items by adjusting their definitions and units. The total number of collected cases was 1288: median age (range), 66 (30–93) years; sex (male/female) 1064/224; pathological type (squamous cell carcinoma, adenocarcinoma, other NSCLC, and unknown) 517, 629, 138, and 4; and stage IIIA and B, 536 and 752. The median overall survival was 26.0 months, with 2-, 5-, and 10-year survival rates of 53.7%, 24.8%, and 15.2%, respectively, in all enrollments. The median progression-free survival was 9.6 months, with 2-, 5-, and 10-year progression-free survival rates of 23.6%, 14.0%, and 9.4%, respectively. Part of the information in the database has been made available on the JLCS web page, and the JLCS members were provided the right to propose research using the database.

**Conclusions:**

The integration and sharing of clinical trial data for research purposes was made real by the nonprofit, academic organization, the JLCS. This database will lead to innovative researches and contribute to the improvement of lung cancer treatment and future research.

## Introduction

Currently, the world is re-evaluating the value of real-world data, which relate to patient health status and the delivery of health care routinely collected from a variety of sources other than traditional clinical trials. The information obtained from the electronic medical records of clinical practice reflects the “real”-world medical situation. Although prospective clinical trials frequently evoke a debate on whether the results can be applied to cases outside the registration criteria, real-world data can fill in the gaps and are undoubtedly of great value. Nevertheless, it is inevitable that the evaluation of efficacy and adverse events in actual clinical practice, for example, the intervals of imaging assessment or the timing of treatment cessation or discontinuation, varies from physician to physician and facility to facility, which can raise concerns regarding the accuracy and reliability of the data. In prospective clinical trials, specific treatments are given to specific subjects according to preset criteria, and the method of evaluating efficacy and toxicity is also predetermined and recorded in detail. Combined with monitoring and auditing, the accuracy and reliability of the data are of distinctive value. Nevertheless, data collected in clinical trials are often left unused after completion of the preplanned analysis. Recently, the release of clinical trial data has been expanding at least partially owing to the willingness of clinical trial participants for data sharing with safeguards in place.^1^ The Pharmaceutical Research and Manufacturers of America and the European Federation of Pharmaceutical Industries and Associations in 2013 and the International Federation of Pharmaceutical Manufacturers & Associations in 2018 made it a principle to disclose and share trial data, including individual data, to researchers; hence, many trial data are now actually disclosed.^2^ For example, Clinical Study Data Requext.com is a clinical trial data-sharing portal site available to multiple companies, and as of February 2022, 13 companies had participated, in which 689 studies were proposed and 533 were approved to date. Nevertheless, only company-accepted studies are eligible, and there is no integration of clinical trial data. In addition, because they are enterprises for profit, it is difficult to avoid certain restrictions.

The sharing of clinical trial data by academic societies, which are nonprofit organizations dedicated to the advancement of health care, has the potential to solve some of the problems that these companies have with data sharing. To share and reuse data from past clinical trials for new research, the Database Committee of the Japan Lung Cancer Society (JLCS) created a database, the JLCS-integrated clinical trial database (JIDB), of existing lung cancer clinical trial data on curative chemoradiotherapy for locally advanced NSCLC. The integration and sharing of clinical trial data with the society’s members, which would have been difficult under the leadership of an individual, a company for profit, or a specific clinical trial group owing to their conflicting interests, was made real by the impartial and neutral organization of the JLCS coming to the fore. In addition, because no external funds were used, a fair and open database was established, and the right to propose research using this database was made available to all members of the JLCS. We believe that the establishment of a clinical trial database led by such an academic society and its sharing with the society’s members are unique, and here, the advantages and problems that emerged from the experience and process of implementation will be reported.

## Materials and Methods

### Subjects

As the first project in the creation of the JIDB, we collected data from randomized phase 2 or 3 trials of radical, concurrent chemoradiation therapy in locally advanced NSCLC conducted in Japan. The formula used to extract trials from EMBASE and MEDLINE databases is found in Supplementary Table 1. From the 200 trials that were detected by the formula in May 2019, trials that met the criteria for the JIDB were selected. Specifically, we excluded 114 studies that did not include locally advanced NSCLC, 52 reports that were not randomized phase 2 or 3 trials, four reports that were subset analyses, five studies that were not designed to evaluate treatment efficacy, two reports that were studies of preoperative treatment, two reports that were designed to evaluate radiation methods, and nine reports that were not conducted by the Japanese Clinical Trial Group. These selections were made in consultation with YO and KI and reviewed by the JLCS Database Committee. The final decisions were made by YN. In July 2020, the search was performed again in the same way to add trials from May 2019 to July 2020.

### Methods

The JLCS is a nonprofit organization established in 1960 with the aim of promoting the advancement of research and the dissemination of knowledge on lung cancer and related areas, thereby contributing to the promotion of the health and welfare of patients, and all mankind. As of April 2021, the number of members of the JLCS was 7367, and the president of the society was Akihiko Gemma. The Database Committee is a subordinate organization chaired by Nobuyuki Yamamoto. From July 2019, the Database Committee requested the representatives of the clinical trial groups, Okayama Lung Cancer Study Group (OLCSG), North Japan Lung Cancer Group (NJLCG), West Japan Oncology Group (WJOG), and Japan Clinical Oncology Group (JCOG), in writing to provide the subject trial data for database creation and sharing. After obtaining consent, data were sent by encrypted USB memory to the JLCS. The addition of the two new trials proceeded in the same way as in September 2020. Consent for data provision was obtained from the Thoracic Oncology Research Group (TORG) and Dr. Seiji Niho, the principal investigator of the SPECTRA trial. Consent for data collection was obtained for all trials in which data provision was requested. All these trials were conducted only in Japan.

### Ethics Approval

The data for which secondary use is envisioned in this plan are information collected through trials conducted in each clinical trial group. All collected data were anonymized by the participating sites and sent to the data centers of each clinical trial, which did not possess correspondence tables, thus making it impossible to identify individuals and obtain individual consent. Therefore, these fall under the category of data that are “anonymized and cannot be used to identify specific individuals,” in which case, Japanese “Ethical Guidelines for Medical Research Involving Human Subjects” stipulates that information can be provided without procedures for obtaining consent. In contrast, because the data to be collected in this study are personal information and need to be taken care for secondary use, information regarding the conduct of this database-creating study and data collection has been disclosed on the website of the JLCS to ensure that participants have the right not to participate in the study.

In November 2019, the Research Ethics Committee of Wakayama Medical University reviewed and approved the accumulation of data and the creation of a database from six trials (no. 2734). In accordance with the bylaws of the clinical trial groups, the JCOG underwent additional review at the National Cancer Center Hospital and received approval from the Research Ethics Committee of Wakayama Medical University. The OLCSG received additional review and approval from the Ethics Committee of Okayama University. To add two new trials, a revised review was performed by the Research Ethics Committee of Wakayama Medical University and received approval in October 2020.

### Statistical Analyses

Statistical analyses were performed using the information collected from the database. Overall survival was defined as the period from the date of enrollment to the date of last observation or death, and progression-free survival (PFS) was defined as the period from the date of enrollment to the date of last observation or the date of confirmed disease progression or death. Each curve was calculated using the Kaplan-Meier method. Statistical analyses were performed using JMP version 14.0 (SAS Inc., Cary, NC).

## Results

### Selection of Clinical Trials

As a result of the first search, eight reports met the criteria, and these reports were related to the following six trials that were selected for inclusion: JCOG9812, a randomized phase 3 study of chest radiation alone versus low-dose daily carboplatin with concurrent thoracic radiotherapy in elderly patients with unresectable locally advanced NSCLC^3^; JCOG0301, a randomized phase 3 study of thoracic radiotherapy with or without daily low-dose carboplatin in elderly patients with NSCLC (modified from JCOG9812)^4^^,^^5^; NJLCG0601, a randomized phase 2 trial of uracil tegafur and cisplatin versus vinorelbine and cisplatin with concurrent thoracic radiotherapy for locally advanced, unresectable, stage III NSCLC^6^; OLCSG0007, a randomized phase 3 trial of docetaxel and cisplatin versus mitomycin, vindesine, and cisplatin with concurrent thoracic radiotherapy in locally advanced NSCLC^7^; WJTOG0105, a randomized phase 3 study comparing second- and third-generation regimens with concurrent thoracic radiotherapy in patients with unresectable, stage III NSCLC^8^^,^^9^; and WJOG5008L, a randomized phase 2 trial of S-1 and cisplatin versus vinorelbine and cisplatin with concurrent thoracic radiotherapy for unresectable, locally advanced NSCLC.^10^ Subsequently, two trials, SPECTRA, a randomized phase 2 study of chemoradiotherapy with S-1 and cisplatin versus pemetrexed and cisplatin for locally advanced nonsquamous NSCLC,^11^ and TORG1018, a randomized phase 2 trial of S-1 and cisplatin versus docetaxel and cisplatin with concurrent thoracic radiotherapy for inoperable stage III NSCLC,^12^ were added as a result of the second search. The details of these eight trials are presented in Table 1. The total number of cases was 1288. For the JCOG0301 and WJOG0105 trials, additional data were collected after long-term follow-up and were reported in 2018 and 2021, respectively^5^^,^^9^; thus, for these two trials, the updated data were included in the JIDB.

**Table 1:** Clinical Trials Included in the Database

Trials	Phase	Enrollment Criteria	Enrollment Period	No. of Cases	Concurrent Chemotherapy	Radiotherapy
JCOG9812^3^	III	Unresectable stage III NSCLC, ≥71 y	November 1999–February 2001	23	Carboplatin 30 mg/m^2^ per d, 5 d a week for 20 d	60 Gy in 30 fractions
				23	None (radiotherapy alone)	
JCOG0301^4^^,^^5^	III	Unresectable stage III NSCLC, ≥71 y	September 2003–March 2010	100	Carboplatin 30 mg/m^2^ per d, 5 d a week for 20 d	60 Gy in 30 fractions
				100	None (radiotherapy alone)	
NJLCG0601^6^	II	Unresectable stage III NSCLC, ≤75 y	February 2006–May 2009	35	Cisplatin 80 mg/m^2^ on d 8 and 36 + tegafur uracil 400 mg/m^2^ on d 1–14 and 29–42	60 Gy in 30 fractions
				31	Cisplatin 80 mg/m^2^ on d 1 and 29 + vinorelbine 20 mg/m^2^ on d 1, 8, 29, and 36	
OLCSG0007^7^	III	Unresectable stage III NSCLC, ≤75 y	July 2000–July 2005	99	Cisplatin 40 mg/m^2^ + docetaxel 40 mg/m^2^, on d 1, 8, 29, and 36	60 Gy in 30 fractions
				101	Cisplatin 80 mg/m^2^ on d 1 and 29 + mitomycin 8 mg/m^2^ + vindesine 3 mg/m^2^, on d 1, 8, 29, and 36	
SPECTRA^11^	II	Unresectable stage III, nonsq, NSCLC, ≤75 y	January 2013–October 2016	52	Cisplatin 60 mg/m^2^ on d 1 + tegafur gimeracil, oteracil potassium 80 mg/m^2^ on d 1–14, every 4 wk, up to four cycles	60 Gy in 30 fractions
				50	Cisplatin 75 mg/m^2^ + pemetrexed 500 mg/m^2^ on d 1, every 3 wk, up to four cycles	
TORG1018^12^	II	Unresectable stage III NSCLC, ≤75 y	May 2011–August 2014	53	Cisplatin 60 mg/m^2^ on d 1 and 29 + tegafur gimeracil, oteracil potassium 80–120 mg/d on d 1–14 and 29–42. Two more cycles were given triweekly as consolidation therapy	60 Gy in 30 fractions
				53	Cisplatin 80 mg/m^2^ + docetaxel 50 mg/m^2^, on d 1 and 29. Two more cycles were given, triweekly as consolidation therapy.	
WJTOG0105^8^^,^^9^	III	Unresectable stage III NSCLC, ≤75 y	September 2001–September 2005	153	Cisplatin 80 mg/m^2^ on d 1 + mitomycin 8 mg/m^2^ on d 1 + vindesine 3 mg/m^2^ on d 1, 8, every 4 wk, up to four cycles	60 Gy in 30 fractions
				151	Weekly carboplatin + paclitaxel (AUC 2) for 6 wk followed by two courses of carboplatin (AUC 5) + paclitaxel (200 mg/m^2^), on d 1	
				152	Weekly carboplatin (AUC 2) + irinotecan (20 mg/m^2^) for 6 wk followed by two courses of carboplatin (AUC 5) + irinotecan (50 mg/m^2^), on d 1	
WJOG5008L^10^	II	Unresectable stage III NSCLC, ≤75 y	September 2009–September 2012	56	Cisplatin 60 mg/m^2^ on d 1 and 29 + tegafur gimeracil, oteracil potassium 80–120 mg on d 1–14 and 29–42. Two more cycles were given, triweekly as consolidation therapy	60 Gy in 30 fractions
				56	Cisplatin 80 mg/m^2^ on d 1 and 29 + vinorelbine 20 mg/m^2^ on d 1, 8 and 29, 36. Two more cycles were given, triweekly as consolidation therapy	

AUC, area under the plasma concentration-time curve; JCOG, Japan Clinical Oncology Group; NJLCG, North Japan Lung Cancer Group; nonsq, nonsquamous; OLCSG, Okayama Lung Cancer Study Group; WJOG, West Japan Oncology Group; WJTOG, West Japan Thoracic Oncology Group.

### Integration of Clinical Trial Data

The total number of items in the collected data was 3091, which were integrated by item name and type of data, resulting in a total of approximately 408 items. These items are categorized into the following: common items, information at registration, pretreatment report, pretreatment clinical findings, pretreatment blood test results, blood test results during observed period, clinical findings during radiotherapy, clinical findings after radiotherapy, clinical findings during observed period, treatment record, tumor evaluation, discontinuation, termination, and subsequent treatment. Integrated definitions of each item were also created. Items with the same content but different names were standardized to the name of the item that the member of the Database Committee in charge judged to be most appropriate. In cases where the item names were the same but the classification or definition was different, the classifications and definitions that would result in the least loss of data that would not alter the data and would be useful for future analysis were determined through discussions between members of the Database Committee and standardized. For example, the WJOG5008L trial classified lung cancer pathological type into the following five categories: adenocarcinoma, squamous cell carcinoma, adenosquamous cell carcinoma, large cell carcinoma, and others. The JCOG0105 trial classified it into 11 categories, including small cell carcinoma, carcinoid, adenoid cystic carcinoma, mucoepidermoid carcinoma, carcinosarcoma, and unclassifiable carcinoma. In the TORG1018 study, there were only two categories, adenocarcinoma and nonadenocarcinoma, but when nonadenocarcinoma was selected, the details were entered in a descriptive form. When integrating them, we first decided to unify them into four categories, adenocarcinoma, squamous cell carcinoma, NSCLC, and others, and fit all results into these categories. The descriptive data of the TORG1018 test were assessed for all its descriptions and assigned to one of the four categories in the same way.

For adverse events, the worst grade of each and the date of onset were included in the JIDB. All data regarding when the adverse event was recognized were standardized to the weeks that elapsed since the start of treatment. For nonhematological toxicity, the number of items evaluated in a preselected format varied widely depending on the trial, and some studies had many descriptive data in the “others” category. We selected 24 key adverse events, such as dyspnea, pneumonitis, left ventricular systolic dysfunction, pericardial effusion, edema limbs, and dermatitis radiation, and assessed whether descriptive data matched these adverse events in the “others” category. If any, we extracted and integrated the data. Data extraction from descriptive data was also performed for complications, comorbidities, and sites of new lesion appearance. After data extraction and integration, all the data were assessed. Thus, 408 items were included in the JIDB. Supplementary Table 2 lists the names, definitions, and number of available data for each item. All data were stored in the cloud and maintained by Genomedia, Inc. (Tokyo, Japan), the contractor for database creation.

### Clinical Background and Survival Curve of the Cases in the Database

**Figure 1:**
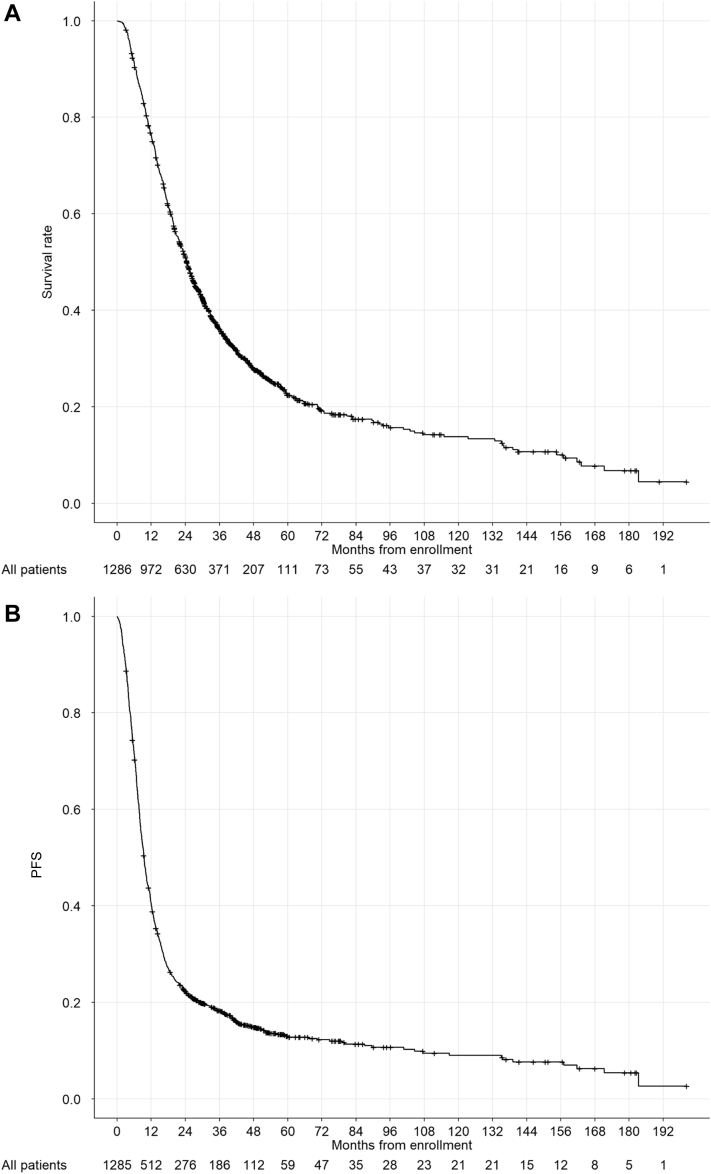
(*A*) PFS and (*B*) survival curve for all patients of the integrated clinical trials. (*A*) The median overall survival was 26.0 months (95% confidence interval: 24.5–27.9), with 2-, 5-, and 10-year survival rates of 53.7%, 24.8%, and 15.2%, respectively, and (*B*) the median PFS was 9.6 months (95% confidence interval: 10.5–22.0), with 2-, 5-, and 10-year PFS rates of 23.6%, 14.0%, and 9.4%, respectively. PFS, progression-free survival.

The background characteristics of the collected data are presented in Table 2. The total number of patients was 1288: median age (range), 66 (30–93) years; sex (male, female), 1064, 224; pathological type (squamous cell carcinoma, adenocarcinoma, other NSCLC, and unknown), 517, 629, 138, and 4; and stage IIIA and B, 536 and 752. The survival and PFS curves using the data from the integrated database are found in Figure 1*A* and *B*. The median overall survival was 26.0 months (95% confidence interval: 24.5–27.9), with 2-, 5-, and 10-year survival rates of 53.7%, 24.8%, and 15.2%, respectively, in all enrollments. The median PFS was 9.6 months (95% confidence interval: 10.5–22.0), with 2-, 5-, and 10-year PFS rates of 23.6%, 14.0%, and 9.4%, respectively. Median observed period was 23.8 (0.3–203) months.

**Table 2:** Clinical Characteristics of the Enrollments

Characteristics	Median (Range)
Age, y	66 (30–93)
Sex	
Male	1064
Female	224
Stage	
IIIA	535
IIIB	752
BSA	1.622 (1.1–2.35)
BMI	21.8 (13.2–52.3)
Pathological type	
Sq	517
Adeno	629
Other NSCLC	138
Unknown	3
Location of primary lesion	
Right upper	389
Right middle	19
Right lower	121
Upper left	276
Lower left	78
Others	33
Smoking, y	40 (1–70)
Smoking, cigarettes	20 (1–100)
Smoking, Brinkman index^a^	945 (1–3680)
T	
0	3
1	172
2	391
3	177
4	476
x	2
N	
0	72
1	53
2	775
3	321
PS	
0	606
1	652
2	10
Irradiated dose, Gy	60 (0–70)
Combined chemotherapy	
Cisplatin, vindesine, mitomicine	254
Cisplatin, tegafur gimeracil oteracil potassium	161
Cisplatin, docetaxel	152
Carboplatin, irinotecan	152
Carboplatin, paclitaxel	151
Carboplatin (daily)	123
Cisplatin, vinorelbine	56
Cisplatin, pemetrexed	50
Vinorelbine	31
Tegafur uracil	35
None	123
Observed period, mo	23.8 (0.3–203)

Adeno, adenocarcinoma; BMI, body mass index; BSA, body surface area; N, lymph node; PS, performance status; Sq, squamous cell carcinoma; T, tumor.

a Brinkman index, the number of cigarettes smoked per day multiplied by the number of years of smoking.

### Data Sharing and Research Solicitation

**Figure 2:**
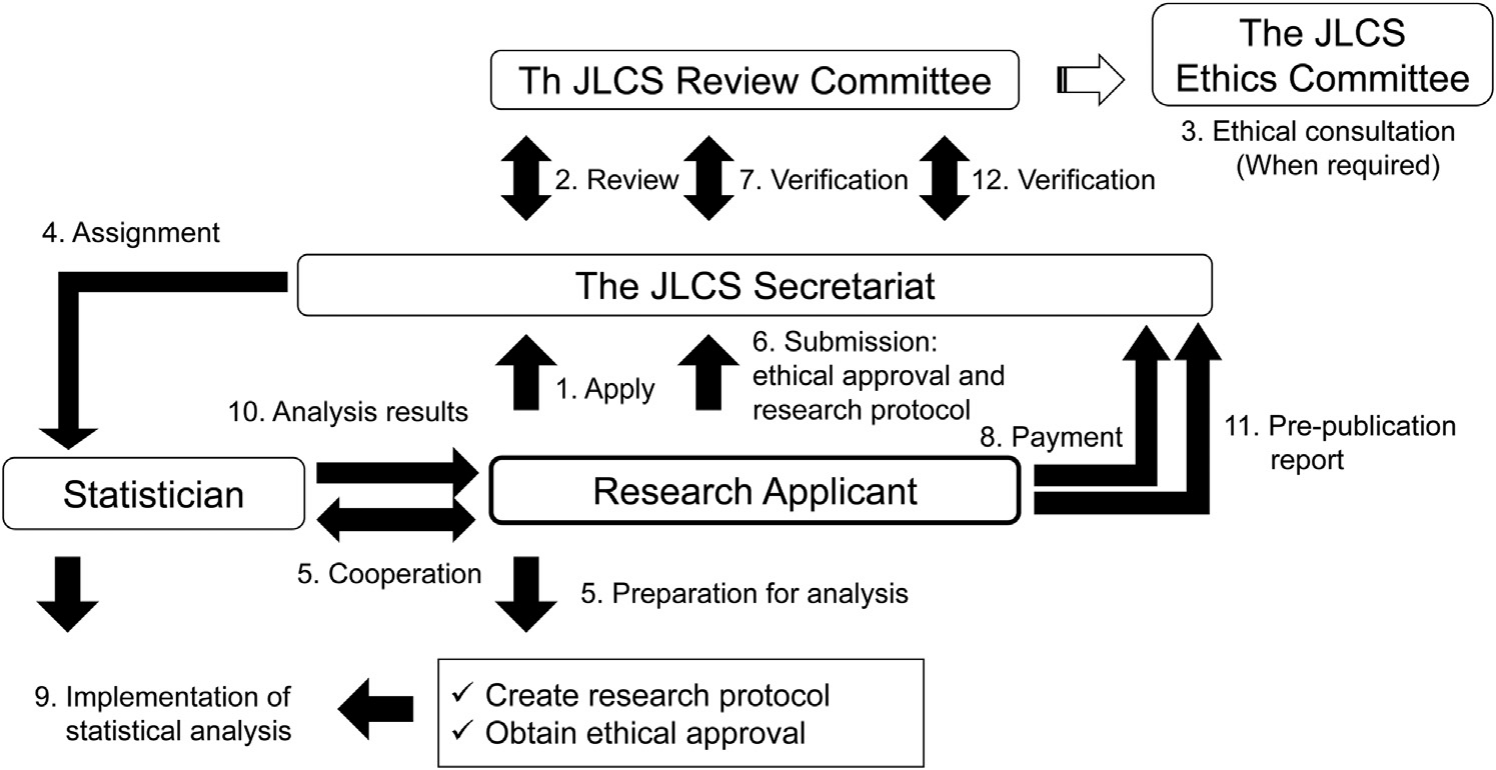
Database-based research proposal and approval process. The JLCS Academic Committee will review all proposals. After approval, the JLCS will appoint a statistician for the analysis. JLCS, Japan Lung Cancer Society.

Information regarding the JIDB, which includes the names of the trials, their treatment, the names and definitions of the items, the number of available cases for each item, and the median and range of age, sex, performance status, pathological type of lung cancer, and clinical stage, will be available on the website of the JLCS. Members of the JLCS will be provided the right to submit research proposals to the JLCS. The Review Committee of the JLCS, which consists of JLCS Academic Committee members and Database Committee members, evaluates the proposals and approves them. Applicants and principal investigators are required to submit an application form along with a letter of intent for the research proposal. After approval, the JLCS will appoint a statistician for the approved study, and the applicant is obligated to bear the actual cost of the statistical analysis. Only appointed statisticians will be allowed to handle the data of the JIDB under a confidentiality agreement and data transfer prohibition agreement. All analyses will be performed on the access-controlled secure cloud service to prevent data dissipation. Before the results of the analysis will be made open to the public, the JLCS must be notified in advance. The JIDB usage will be allowed only for academic purposes and not for commercial purposes. A schema for JIDB usage is found in Figure 2. The database will be released, and a call will be made for research proposals in the second quarter of 2022.

## Discussion

Herein, we received data from eight randomized clinical trials conducted by six clinical trial groups under the initiative of the JLCS, a nonprofit organization, and created a database by integrating these data—JIDB. In addition, the JLCS will grant right of usage of the JIDB to all members of society and solicit research using this database.

To the best of our knowledge, society-led database construction and data-sharing systems with society members are unprecedented in the world. This first project included randomized phase 2 or 3 trials of curative chemoradiotherapy for locally advanced NSCLC. The reasons for targeting this stage were as follows: first, despite the completion of several large clinical trials, there have been no reports of integrated analysis, especially the integration of individual data. In fact, many aspects of curative chemoradiotherapy, such as factors to predict efficacy and adverse events, remain unexplored; hence, there is considerable room for the JIDB to contribute. In addition, the importance of this population has been increasing since the PACIFIC trial revealed the benefit of maintenance therapy with durvalumab, programmed death-ligand 1 (PD-L1) inhibitor, after concurrent curative chemoradiotherapy completion in 2017.^13^ In this population, several new clinical trials are underway, including trials on PD-1 or PD-L1 inhibitors in combination with chemotherapy and radiation therapy concurrently.^14^ Researches, such as those for the predictors of efficacy and adverse events, such as pneumonitis, for the comparison of combined regimens, or for the prognostic factors at the time of treatment initiation, are expected to contribute to the development of new therapies in the future.

This JIDB has a highly reliable data set. We excluded single-arm studies from the scope of this database because there were many studies of various sizes and purposes; hence, it was difficult to objectively identify sufficiently reliable trials from among them. Randomized trials have balanced prognostic factors that other trials are unable to achieve. Furthermore, because randomized trials require a certain number of cases, an experienced support system, and sufficient resources, we determined that the reliability of the data could be enhanced by making it a condition for data collection that the trials be randomized. As a result, six clinical trial groups that conducted the eight trials were included in the JIDB. All clinical trial groups have abundant experience and strong achievements, The JCOG Lung Cancer Study Group reported results of four phase 3 trials between 2014 and 2016,^15^, ^16^, ^17^, ^18^ and the WJOG also reported results of four phase 3 trials targeting lung cancer in the past 5 years.^19^, ^20^, ^21^, ^22^ The NJLCG and TORG have reported four^23^, ^24^, ^25^, ^26^ and three^12^^,^^27^^,^^28^ randomized phase 2 trials, respectively, and several other single-arm trials in the past 5 years. The National Cancer Center Hospital EAST was the lead site of the SPECTRA trial, and the primary investigator, Niho Seiji, has abundant experience from many clinical trials,^29^, ^30^, ^31^, ^32^, ^33^ which made it possible to constitute a reliable data set. In all trials, the method of evaluating efficacy was specified in detail, including the interval and method of imaging tests; the method of evaluating efficacy and adverse events was also specified in advance, to be based on the Response Evaluation Criteria in Solid Tumors and the Common Terminology Criteria for Adverse Events. Furthermore, in addition to the large number of assessable patients (1288), JIDB also included data from follow-up analyses in WJTOG0105 and JCOG0301,^5,9^ which were collected 11 and 6 years after the initial reports, respectively. Availability for analysis of such long-term observed patients is also a strong point of this JIDB.

In two phase 3 trials for locally advanced-stage NSCLC reported in 2016 and 2017, both being implemented outside of Japan, median survival time was 20.7 to 23.3 months and 25.0 to 26.8 months, respectively, and median PFS was 12.0 to 14.0 months and 9.8 to 11.4 months, respectively.^34^^,^^35^ From the results of the present JIDB, median survival time and PFS are 26 months and 9.6 months, respectively, comparable with former studies, which indicates the universality of the JIDB. The current standard of care is concurrent chemoradiotherapy followed by durvalumab, and the efficacy and safety of chemoradiotherapy and the factors related to them are of high interest.

The most difficult part in creating this database was the integration of data. The trials included spanned a wide range of time periods, from 2005 to 2018, and each trial consisted of a different clinical trial group. Thus, the collected information was relatively diverse. More than 3000 items were initially integrated based on their names, but units and definitions (classifications) were also needed to be simultaneously unified. It was also difficult to handle descriptive data in this study. The items considered important were extracted, but such a task required the confirmation of a physician with deep knowledge and experience of lung cancer treatment and clinical trials. On the basis of this experience, future clinical trials should be conducted to standardize the names of items and their definitions between trials, with a view to building databases and reuse for future research.

In addition to maximizing the value of clinical trial data and generating new knowledge through its reuse, providing the JLCS members with opportunities to use large-scale data and contributing to the development of researchers is one of the marked values of building database and sharing with the JLCS members. In contrast, we do not disclose the data itself to the researchers, but only the names of data items, the number of available cases for each item, and their definitions; furthermore, only statisticians appointed by JLCS were allowed to handle and analyze the data, and all data viewing and analysis can only be performed on the cloud system. Data integrity is one of the most important aspects of building and releasing such databases, and it has been and will continue to be crucial to pay close attention to it.

As a limitation, there are only a limited number of items for which all trials had data, and this may limit the analysis, although the accumulated number of cases was 1288. In addition, owing to the wide range of years of the trials, there were little data available for analysis, such as the *EGFR* or *ALK* gene alterations or PD-L1 expression, or the treatment with tyrosine kinase inhibitors or PD-1, PD-L1 inhibitors. The versions of the Common Terminology Criteria for Adverse Events used to assess toxicity severity in all included trials varied from 2.0 to 5.0, limiting the significance of the integrated assessment of adverse events (Supplementary Table 3). Finally, the included clinical trials were limited to those conducted in Japan. The reason for this is that the first round of database creation targeted the population that was likely to understand and support the significance of database creation by the JLCS. For the second and subsequent projects, we would like to collaborate with other countries to create a database on a global scale.

The creation of a database of clinical trials will enable the reuse of data with a higher degree of accuracy than real-world data, and this may help to conduct new and promising clinical trials and eliminate wasteful clinical trials in the future. In addition, the sharing of data among society members will lead to the discovery of innovative research proposals and even lead to the discovery of young talented clinical researchers. Thus, we are confident that the JIDB will contribute to the improvement of lung cancer treatment and future research.

## CRediT Authorship Contribution Statement

**Yuichi Ozawa:** Conceptualization, Methodology, Investigation, Analysis, Data curation, Writing - original draft.

**Nobuyuki Yamamoto**: Conceptualization, Methodology, Manuscript review, Supervision, Project administration.

**Kouji Yamamoto:** Methodology, Analysis, Validation, Manuscript review.

**Kentaro Ito:** Methodology, Analysis, Manuscript review.

**Hirotsugu Kenmotsu, Hidetoshi Hayashi, Takehito Shukuya, Daichi Fujimoto:** Methodology, Manuscript review.

**Shunichi Sugawara, Seiji Niho, Yuichiro Ohe, Hiroaki Okamoto, Kazuhiko Nakagawa, Katsuyuki Kiura, Ichiro Yoshino:** Data provision, Manuscript review.

**Akihito Gemma:** Funding acquisition, Supervision, Manuscript review, Project administration.
