# Supplementary material for: Creation of an Integrated Clinical Trial Database and Data Sharing for Conducting New Research by the Japan Lung Cancer Society
Source: JTO Clin Res Rep. 2022 Mar 27;3(5):100317. doi: 10.1016/j.jtocrr.2022.100317 (PMC9048121; doi:10.1016/j.jtocrr.2022.100317)
Supplement: Supplementary Table 1-3 [file mmc1.docx]

**Table S1. Formula used to extract trials from EMBASE and MEDLIN**

(((('lung cancer'/exp OR 'lung tumor'/exp OR 'lung carcinoma'/exp OR 'lung carcinoma'/exp) AND ('non small cell':ab,ti OR 'non small cell':ab,ti)) OR 'non small cell lung cancer'/exp OR 'nsclc':ab,ti) AND ((stage3 OR stage) AND iii OR 'locally advanced non-small cell lung cancer' OR unresectable) AND ('japan' OR 'japanese (people)')) AND ('phase 2 clinical trial' OR 'phase 3 clinical trial' OR 'randomized controlled trial')

**Table S2.** **Definition, name, and number of available cases for each item**

| Items | Number of cases | Definition |
| --- | --- | --- |
| **PRE-TREATMENT -ALL-** | | |
| Enrollment date | 1288 | Date |
| Sex | 1288 | M, F |
| Age | 1288 | number |
| TNM-T | 1221 | T0, T1, T2, T3, T4, TX |
| TNM-N | 1221 | N0, N1, N2, N3, NX |
| Stage | 1287 | ⅢA, ⅢB |
| Results of randomization | 1288 | Allocated group |
| Hight, cm | 822 | number |
| Weight, kg | 1022 | number |
| Body surface area, m^2^ | 822 | number |
| BMI | 822 | number |
| Smoking history | 1021 | Never, Ex, Current |
| Brinkman index | 892 | number |
| Medical history | 1021 | yes, no |
| Medical history - diabetes mellitus | 1021 | yes, no |
| Medical history - hypertension | 1021 | Text |
| Medical history - arrhythmia | 1021 | yes, no |
| Medical history - COPD | 775 | yes, no |
| Medical history - pulmonary emphysema | 775 | yes, no |
| Medical history - bronchial asthma | 775 | yes, no |
| Medical history - others | 1021 | yes, no |
| Medical history - text data about others | 405 | Text |
| Weight loss of ≥5% within 6 months | 1004 | yes, no |
| Location of primary site - right upper lobe | 915 | yes, no |
| Location of primary site - right middle lobe | 915 | yes, no |
| Location of primary site - right lower lobe | 915 | yes, no |
| Location of primary site - left upper lobe | 915 | yes, no |
| Location of primary site - left lower lobe | 915 | yes, no |
| Location of primary site - others | 915 | yes, no |
| Location of primary site -text data about others | 33 | text |
| Histological classification of the cancer | 1287 | adeno, squamous, other NSCLC, others, unknown |
| Histological classification of the cancer, detail about others | 9 | text |
| Histological classification - text data about others | 85 | text |
| EGFR mutation test results | 274 | positive, negative, unknown |
| EGFR mutation test results - text data | 36 | text |
| Performance status, pre-treatment | 1268 | 0, 1, 2 |
| Performance status evaluation date | 108 | date |
| Test date, complete blood count | 910 | date |
| White blood cell, /mm^3^ | 911 | number |
| Neutrophil, /mm^3^ | 909 | number |
| Hemoglobin, g/dl | 911 | number |
| Platelet, /mm^3^ | 911 | number |
| Red blood cell, /mm^3^ | 499 | number |
| Test date, biochemistry | 911 | date |
| Total bilirubin, mg/dl | 911 | number |
| Aspartate aminotransferase, IU/l | 911 | number |
| Alanine aminotransferase, IU/l | 911 | number |
| Creatinine, mg/dl | 911 | number |
| Total protein, g/dl | 804 | number |
| Blood urea nitrogen, mg/dl | 805 | number |
| Albumin, g/dl | 908 | number |
| Sodium, mEq/l | 910 | number |
| Alkaline phosphatase, IU/l | 704 | number |
| Potassium, mEq/l | 910 | number |
| Lactate dehydrogenase, IU/l | 803 | number |
| Chloride, IU/l | 603 | number |
| Calcium, mg/dl | 897 | number |
| Test date, creatinine clearance | 152 | number |
| creatinine clearance, ml/min | 152 | number |
| Calculation method; creatinine clearance | 36 | Cock-croft, 24-hour urine collection |
| Test date, c-reactive protein | 805 | date |
| C-reactive protein, mg/dl | 802 | number |
| Test date, arterial blood gas | 801 | date |
| Partial pressure of arterial oxygen, torr | 802 | number |
| Partial pressure of arterial carbon dioxide, torr | 801 | number |
| Test date, Sialylated Carbohydrate Antigen KL-6 | 538 | date |
| Sialylated carbohydrate antigen KL-6, IU/l | 533 | number |
| Test date, electrocardiogram | 302 | date |
| Abnormal findings of electrocardiogram | 344 | yes, no, unknown |
| Text data about Abnormal findings of electrocardiogram | 69 | text |
| **AFTER TRIAL TREATMENT START -LABORATORY DATA-** | | |
| Date of the lowest white blood cell value | 889 | date |
| CTCAE grade of white blood cell decrease | 1260 | 0, 1, 2, 3, 4 |
| Date of the lowest hemoglobin value | 890 | date |
| CTCAE grade of hemoglobin decrease | 1062 | 0, 1, 2, 3, 4 |
| Date of the lowest platelet value | 884 | date |
| CTCAE grade of platelet decrease | 1252 | 0, 1, 2, 3, 4 |
| Date of the lowest neutrophil value | 889 | date |
| CTCAE grade of neutrophil decrease | 1259 | 0, 1, 2, 3, 4 |
| Date of the lowest red blood cell value | 440 | date |
| CTCAE grade of red blood cell decrease | 440 | 0, 1, 2, 3, 4 |
| Date of the lowest total protein value | 776 | date |
| CTCAE grade of total protein decrease | 776 | number |
| Date of the lowest albumin value | 881 | date |
| CTCAE grade of albumin decrease | 881 | 0, 1, 2, 3, 4 |
| Date of the highest total bilirubin value | 866 | date |
| CTCAE grade of total bilirubin decrease | 971 | 0, 1, 2, 3, 4 |
| Date of the highest aspartate aminotransferase | 870 | date |
| CTCAE grade of aspartate aminotransferase | 975 | 0, 1, 2, 3, 4 |
| Date of the highest alanine aminotransferase | 870 | date |
| CTCAE grade of alanine aminotransferase increase | 976 | 0, 1, 2, 3, 4 |
| Date of the highest lactate dehydrogenase | 781 | date |
| CTCAE grade of lactate dehydrogenase increase | 781 | number |
| Date of the highest creatinine | 868 | date |
| CTCAE grade of creatinine increase | 974 | 0, 1, 2, 3, 4 |
| Date of the highest blood urea nitrogen | 782 | date |
| CTCAE grade of blood urea nitrogen | 782 | number |
| Date of the highest alkaline phosphatase | 589 | date |
| CTCAE grade of alkaline phosphatase increase | 589 | 0, 1, 2, 3, 4 |
| Date of the lowest chloride | 588 | date |
| CTCAE Grade of chloride decrease | 588 | number |
| Date of the highest sodium value | 866 | date |
| CTCAE Grade of sodium increase | 866 | 0, 1, 2, 3, 4 |
| Date of the lowest sodium | 884 | date |
| CTCAE grade of sodium decrease | 884 | 0, 1, 2, 3, 4 |
| Date of the highest potassium | 869 | date |
| CTCAE grade of potassium increase | 869 | 0, 1, 2, 3, 4 |
| Date of the lowest potassium | 870 | date |
| CTCAE grade of potassium decrease | 870 | 0, 1, 2, 3, 4 |
| Date of the highest calcium | 792 | date |
| CTCAE grade of calcium increase | 792 | 0, 1, 2, 3, 4 |
| Date of the lowest calcium | 807 | date |
| CTCAE grade of calcium decrease | 807 | 0, 1, 2, 3, 4 |
| Date of the lowest oxygen saturation | 236 | date |
| The lowest value of oxygen saturation | 236 | number |
| Date of the lowest partial pressure of arterial oxygen | 508 | date |
| The lowest value of partial pressure of arterial oxygen | 509 | number |
| Date of the lowest partial pressure of arterial carbon dioxide | 400 | date |
| The lowest value of partial pressure of arterial carbon dioxide | 401 | number |
| **AFTER TRIAL TREATMENT START TO THE END – CLINICAL FINDINGS-** | | |
| Worst performance status during trial treatment | 644 | 0, 1, 2, 3, 4 |
| Time when worst performance status was observed during trial treatment (weeks from trial treatment start), weeks | 532 | number |
| Worst CTCAE grade of dyspnea during trial treatment | 886 | 0, 1, 2, 3, 4, 5 |
| Time when worst CTCAE grade of dyspnea was observed (weeks from trial treatment start) during trial treatment, weeks | 690 | number |
| Worst CTCAE grade of pneumonitis during trial treatment | 977 | 0, 1, 2, 3, 4, 5 |
| Time when worst CTCAE grade of pneumonitis was observed (weeks from trial treatment start) during trial treatment, weeks | 685 | number |
| Worst CTCAE grade of left ventricular systolic dysfunction during trial treatment | 45 | 0, 1, 2, 3, 4, 5 |
| Time when worst CTCAE grade of left ventricular systolic dysfunction was observed (weeks from trial treatment start) during trial treatment, weeks | 45 | number |
| Worst CTCAE grade of pericardial effusion during trial treatment | 341 | 0, 1, 2, 3, 4, 5 |
| Time when worst CTCAE grade of pericardial effusion was observed (weeks from trial treatment start) during trial treatment, weeks | 239 | number |
| Worst CTCAE grade of edema limbs during trial treatment | 347 | 0, 1, 2, 3, 4, 5 |
| Time when worst CTCAE grade of edema limbs was observed (weeks from trial treatment start) during trial treatment, weeks | 241 | number |
| Worst CTCAE grade of fatigue, malaise, or lethargy during trial treatment | 902 | 0, 1, 2, 3, 4, 5 |
| Time when worst CTCAE grade of fatigue, malaise, or lethargy was observed (weeks from trial treatment start) during trial treatment, weeks | 769 | number |
| Worst CTCAE grade of fever during trial treatment | 982 | 0, 1, 2, 3, 4, 5 |
| Time when worst CTCAE grade of fever was observed (weeks from trial treatment start) during trial treatment, weeks | 725 | number |
| Worst CTCAE grade of dermatitis radiation during trial treatment | 905 | 0, 1, 2, 3, 4, 5 |
| Time when worst CTCAE grade of dermatitis radiation was observed (weeks from trial treatment start) during trial treatment, weeks | 832 | number |
| Worst CTCAE grade of anorexia during trial treatment | 502 | 0, 1, 2, 3, 4, 5 |
| Time when worst CTCAE grade of anorexia was observed (weeks from trial treatment start) during trial treatment, weeks | 348 | number |
| Worst CTCAE grade of dysphagia during trial treatment | 786 | 0, 1, 2, 3, 4, 5 |
| Time when worst CTCAE grade of dysphagia was observed (weeks from trial treatment start) during trial treatment, weeks | 739 | number |
| Worst CTCAE grade of nausea during trial treatment | 989 | 0, 1, 2, 3, 4, 5 |
| Time when worst CTCAE grade of nausea was observed (weeks from trial treatment start) during trial treatment, weeks | 815 | number |
| Worst CTCAE grade of vomiting during trial treatment | 986 | 0, 1, 2, 3, 4, 5 |
| Time when worst CTCAE grade of vomiting was observed (weeks from trial treatment start) during trial treatment, weeks | 711 | number |
| Worst CTCAE grade of febrile neutropenia during trial treatment | 988 | 0, 1, 2, 3, 4, 5 |
| Time when worst CTCAE grade of febrile neutropenia was observed (weeks from trial treatment start) during trial treatment, weeks | 693 | number |
| Worst CTCAE grade of cough during trial treatment | 343 | 0, 1, 2, 3, 4, 5 |
| Time when worst CTCAE grade of cough was observed (weeks from trial treatment start) during trial treatment, weeks | 245 | number |
| Worst CTCAE grade of diarrhea during trial treatment | 747 | 0, 1, 2, 3, 4, 5 |
| Time when worst CTCAE grade of diarrhea was observed (weeks from trial treatment start) during trial treatment, weeks | 477 | number |
| Worst CTCAE grade of constipation during trial treatment | 752 | 0, 1, 2, 3, 4, 5 |
| Time when worst CTCAE grade of constipation was observed (weeks from trial treatment start) during trial treatment, weeks | 593 | number |
| Worst CTCAE grade of infection during trial treatment | 841 | 0, 1, 2, 3, 4, 5 |
| Time when worst CTCAE grade of infection was observed (weeks from trial treatment start) during trial treatment, weeks | 654 | number |
| Worst CTCAE grade of mucositis oral and pharyngeal mucositis during trial treatment | 750 | 0, 1, 2, 3, 4, 5 |
| Time when worst CTCAE grade of mucositis oral and pharyngeal mucositis was observed (weeks from trial treatment start) during trial treatment, weeks | 474 | number |
| Worst CTCAE grade of peripheral motor neuropathy during trial treatment | 752 | 0, 1, 2, 3, 4, 5 |
| Time when worst CTCAE grade of peripheral motor neuropathy was observed (weeks from trial treatment start) during trial treatment, weeks | 445 | number |
| Worst CTCAE grade of peripheral sensory neuropathy during trial treatment | 749 | 0, 1, 2, 3, 4, 5 |
| Time when worst CTCAE grade of peripheral motor neuropathy was observed (weeks from trial treatment start) during trial treatment, weeks | 455 | number |
| Worst CTCAE grade of esophagitis during trial treatment | 255 | 0, 1, 2, 3, 4, 5 |
| Time when worst CTCAE grade of esophagitis was observed (weeks from trial treatment start) during trial treatment, weeks | 165 | number |
| Worst CTCAE grade of alopecia during trial treatment | 305 | 0, 1, 2, 3, 4, 5 |
| Time when worst CTCAE grade of alopecia was observed (weeks from trial treatment start) during trial treatment, weeks | 19 | number |
| Worst CTCAE grade of allergic reaction during trial treatment | 208 | 0, 1, 2, 3, 4, 5 |
| Time when worst CTCAE grade of allergic reaction was observed (weeks from trial treatment start) during trial treatment, weeks | 1 | number |
| Worst CTCAE grade of pulmonary fibrosis during trial treatment | 208 | 0, 1, 2, 3, 4, 5 |
| Time when worst CTCAE grade of pulmonary fibrosis was observed (weeks from trial treatment start) during trial treatment, weeks | 0 | number |
| **AFTER END OF TRIAL TREATMENT TO FINAL OBSERVED DAY – CLINICAL FINDINGS-** | | |
| Worst performance status after trial treatment | 607 | 0, 1, 2, 3, 4 |
| Time when worst performance status was observed after trial treatment (weeks from trial treatment start), weeks | 74 | number |
| Worst CTCAE grade of dyspnea after trial treatment | 838 | 0, 1, 2, 3, 4, 5 |
| Time when worst CTCAE grade of dyspnea was observed (weeks from trial treatment start) after trial treatment, weeks | 210 | number |
| Worst CTCAE grade of pneumonitis after trial treatment | 972 | 0, 1, 2, 3, 4, 5 |
| Time when worst CTCAE grade of pneumonitis was observed (weeks from trial treatment start) after trial treatment, weeks | 328 | number |
| Worst CTCAE grade of left ventricular systolic dysfunction after trial treatment | 0 | 0, 1, 2, 3, 4, 5 |
| Time when worst CTCAE grade of left ventricular systolic dysfunction was observed (weeks from trial treatment start) after trial treatment, weeks | 0 | number |
| Worst CTCAE grade of pericardial effusion after trial treatment | 612 | 0, 1, 2, 3, 4, 5 |
| Time when worst CTCAE grade of pericardial effusion was observed (weeks from trial treatment start) after trial treatment, weeks | 239 | number |
| Worst CTCAE grade of edema limbs after trial treatment | 285 | 0, 1, 2, 3, 4, 5 |
| Time when worst CTCAE grade of edema limbs was observed (weeks from trial treatment start) after trial treatment, weeks | 194 | number |
| Worst CTCAE grade of fatigue, malaise, or lethargy after trial treatment | 819 | 0, 1, 2, 3, 4, 5 |
| Time when worst CTCAE grade of fatigue, malaise, or lethargy was observed (weeks from trial treatment start) after trial treatment, weeks | 264 | number |
| Worst CTCAE grade of fever after trial treatment | 887 | 0, 1, 2, 3, 4, 5 |
| Time when worst CTCAE grade of fever was observed (weeks from trial treatment start) after trial treatment, weeks | 222 | number |
| Worst CTCAE grade of dermatitis radiation after trial treatment | 836 | 0, 1, 2, 3, 4, 5 |
| Time when worst CTCAE grade of dermatitis radiation was observed (weeks from trial treatment start) after trial treatment, weeks | 281 | number |
| Worst CTCAE grade of anorexia after trial treatment | 452 | 0, 1, 2, 3, 4, 5 |
| Time when worst CTCAE grade of anorexia was observed (weeks from trial treatment start) after trial treatment, weeks | 291 | number |
| Worst CTCAE grade of dysphagia after trial treatment | 698 | 0, 1, 2, 3, 4, 5 |
| Time when worst CTCAE grade of dysphagia was observed (weeks from trial treatment start) after trial treatment, weeks | 219 | number |
| Worst CTCAE grade of nausea after trial treatment | 893 | 0, 1, 2, 3, 4, 5 |
| Time when worst CTCAE grade of nausea was observed (weeks from trial treatment start) after trial treatment, weeks | 285 | number |
| Worst CTCAE grade of after trial treatment | 906 | 0, 1, 2, 3, 4, 5 |
| Time when worst CTCAE grade of vomiting was observed (weeks from trial treatment start) after trial treatment, weeks | 219 | number |
| Worst CTCAE grade of febrile neutropenia after trial treatment | 909 | 0, 1, 2, 3, 4, 5 |
| Time when worst CTCAE grade of febrile neutropenia was observed (weeks from trial treatment start) after trial treatment, weeks | 210 | number |
| Worst CTCAE grade of cough after trial treatment | 300 | 0, 1, 2, 3, 4, 5 |
| Time when worst CTCAE grade of cough was observed (weeks from trial treatment start) after trial treatment, weeks | 204 | number |
| Worst CTCAE grade of diarrhea after trial treatment | 702 | 0, 1, 2, 3, 4, 5 |
| Time when worst CTCAE grade of diarrhea was observed (weeks from trial treatment start) after trial treatment, weeks | 20 | number |
| Worst CTCAE grade of constipation after trial treatment | 683 | 0, 1, 2, 3, 4, 5 |
| Time when worst CTCAE grade of constipation was observed (weeks from trial treatment start) after trial treatment, weeks | 79 | number |
| Worst CTCAE grade of infection after trial treatment | 826 | 0, 1, 2, 3, 4, 5 |
| Time when worst CTCAE grade of infection was observed (weeks from trial treatment start) after trial treatment, weeks | 219 | number |
| Worst CTCAE grade of mucositis oral and pharyngeal mucositis after trial treatment | 710 | 0, 1, 2, 3, 4, 5 |
| Time when worst CTCAE grade of mucositis oral and pharyngeal mucositis was observed (weeks from trial treatment start) after trial treatment, weeks | 21 | number |
| Worst CTCAE grade of peripheral motor neuropathy after trial treatment | 721 | 0, 1, 2, 3, 4, 5 |
| Time when worst CTCAE grade of peripheral motor neuropathy was observed (weeks from trial treatment start) after trial treatment, weeks | 8 | number |
| Worst CTCAE grade of peripheral sensory neuropathy after trial treatment | 714 | 0, 1, 2, 3, 4, 5 |
| Time when worst CTCAE grade of peripheral motor neuropathy was observed (weeks from trial treatment start) after trial treatment, weeks | 17 | number |
| Worst CTCAE grade of esophagitis after trial treatment | 485 | 0, 1, 2, 3, 4, 5 |
| Time when worst CTCAE grade of esophagitis was observed (weeks from trial treatment start) after trial treatment, weeks | 92 | number |
| Worst CTCAE grade of alopecia after trial treatment | 281 | 0, 1, 2, 3, 4, 5 |
| Time when worst CTCAE grade of alopecia was observed (weeks from trial treatment start) after trial treatment, weeks | 27 | number |
| Worst CTCAE grade of allergic reaction after trial treatment | 177 | 0, 1, 2, 3, 4, 5 |
| Time when worst CTCAE grade of allergic reaction was observed (weeks from trial treatment start) after trial treatment, weeks | 0 | number |
| Worst CTCAE grade of pulmonary fibrosis after trial treatment | 192 | 0, 1, 2, 3, 4, 5 |
| Time when worst CTCAE grade of pulmonary fibrosis was observed (weeks from trial treatment start) after trial treatment, weeks | 4 | number |
| **DURING THE WHOLE OBSERVED PERIOD -CLINICAL FINDINGS-** | | |
| Worst performance status during the whole observed period | 650 | 0, 1, 2, 3, 4 |
| Time when worst performance status was observed during the whole observed period (weeks from trial treatment start), weeks | 468 | number |
| Worst CTCAE grade of dyspnea during the whole observed period | 892 | 0, 1, 2, 3, 4, 5 |
| Time when worst CTCAE grade of dyspnea was observed (weeks from trial treatment start) during the whole observed period, weeks | 652 | number |
| Worst CTCAE grade of pneumonitis during the whole observed period | 1203 | 0, 1, 2, 3, 4, 5 |
| Time when worst CTCAE grade of pneumonitis was observed (weeks from trial treatment start) during the whole observed period, weeks | 565 | number |
| Worst CTCAE grade of left ventricular systolic dysfunction during the whole observed period | 45 | 0, 1, 2, 3, 4, 5 |
| Time when worst CTCAE grade of left ventricular systolic dysfunction was observed (weeks from trial treatment start) during the whole observed period, weeks | 45 | number |
| Worst CTCAE grade of pericardial effusion during the whole observed period | 615 | 0, 1, 2, 3, 4, 5 |
| Time when worst CTCAE grade of pericardial effusion was observed (weeks from trial treatment start) during the whole observed period, weeks | 241 | number |
| Worst CTCAE grade of edema limbs during the whole observed period | 347 | 0, 1, 2, 3, 4, 5 |
| Time when worst CTCAE grade of edema limbs was observed (weeks from trial treatment start) during the whole observed period, weeks | 241 | number |
| Worst CTCAE grade of fatigue, malaise, or lethargy during the whole observed period | 927 | 0, 1, 2, 3, 4, 5 |
| Time when worst CTCAE grade of fatigue, malaise, or lethargy was observed (weeks from trial treatment start) during the whole observed period, weeks | 713 | number |
| Worst CTCAE grade of fever during the whole observed period | 995 | 0, 1, 2, 3, 4, 5 |
| Time when worst CTCAE grade of fever was observed (weeks from trial treatment start) during the whole observed period, weeks | 704 | number |
| Worst CTCAE grade of dermatitis radiation during the whole observed period | 912 | 0, 1, 2, 3, 4, 5 |
| Time when worst CTCAE grade of dermatitis radiation was observed (weeks from trial treatment start) during the whole observed period, weeks | 812 | number |
| Worst CTCAE grade of anorexia during the whole observed period | 576 | 0, 1, 2, 3, 4, 5 |
| Time when worst CTCAE grade of anorexia was observed (weeks from trial treatment start) during the whole observed period, weeks | 361 | number |
| Worst CTCAE grade of dysphagia during the whole observed period | 794 | 0, 1, 2, 3, 4, 5 |
| Time when worst CTCAE grade of dysphagia was observed (weeks from trial treatment start) during the whole observed period, weeks | 726 | number |
| Worst CTCAE grade of nausea during the whole observed period | 995 | 0, 1, 2, 3, 4, 5 |
| Time when worst CTCAE grade of nausea was observed (weeks from trial treatment start) during the whole observed period, weeks | 773 | number |
| Worst CTCAE grade of vomiting during the whole observed period | 1061 | 0, 1, 2, 3, 4, 5 |
| Time when worst CTCAE grade of vomiting was observed (weeks from trial treatment start) during the whole observed period, weeks | 697 | number |
| Worst CTCAE grade of febrile neutropenia during the whole observed period | 1061 | 0, 1, 2, 3, 4, 5 |
| Time when worst CTCAE grade of febrile neutropenia was observed (weeks from trial treatment start) during the whole observed period, weeks | 681 | number |
| Worst CTCAE grade of cough during the whole observed period | 347 | 0, 1, 2, 3, 4, 5 |
| Time when worst CTCAE grade of cough was observed (weeks from trial treatment start) during the whole observed period, weeks | 254 | number |
| Worst CTCAE grade of diarrhea during the whole observed period | 822 | 0, 1, 2, 3, 4, 5 |
| Time when worst CTCAE grade of diarrhea was observed (weeks from trial treatment start) during the whole observed period, weeks | 449 | number |
| Worst CTCAE grade of constipation during the whole observed period | 756 | 0, 1, 2, 3, 4, 5 |
| Time when worst CTCAE grade of constipation was observed (weeks from trial treatment start) during the whole observed period, weeks | 560 | number |
| Worst CTCAE grade of infection during the whole observed period | 921 | 0, 1, 2, 3, 4, 5 |
| Time when worst CTCAE grade of infection was observed (weeks from trial treatment start) during the whole observed period, weeks | 633 | number |
| Worst CTCAE grade of mucositis oral and pharyngeal mucositis during the whole observed period | 822 | 0, 1, 2, 3, 4, 5 |
| Time when worst CTCAE grade of mucositis oral and pharyngeal mucositis was observed (weeks from trial treatment start) during the whole observed period, weeks | 458 | number |
| Worst CTCAE grade of peripheral motor neuropathy during the whole observed period | 756 | 0, 1, 2, 3, 4, 5 |
| Time when worst CTCAE grade of peripheral motor neuropathy was observed (weeks from trial treatment start) during the whole observed period, weeks | 426 | number |
| Worst CTCAE grade of peripheral sensory neuropathy during the whole observed period | 822 | 0, 1, 2, 3, 4, 5 |
| Time when worst CTCAE grade of peripheral motor neuropathy was observed (weeks from trial treatment start) during the whole observed period, weeks | 382 | number |
| Worst CTCAE grade of esophagitis during the whole observed period | 769 | 0, 1, 2, 3, 4, 5 |
| Time when worst CTCAE grade of esophagitis was observed (weeks from trial treatment start) during the whole observed period, weeks | 213 | number |
| Worst CTCAE grade of alopecia during the whole observed period | 381 | 0, 1, 2, 3, 4, 5 |
| Time when worst CTCAE grade of alopecia was observed (weeks from trial treatment start) during the whole observed period, weeks | 32 | number |
| Worst CTCAE grade of allergic reaction during the whole observed period | 208 | 0, 1, 2, 3, 4, 5 |
| Time when worst CTCAE grade of allergic reaction was observed (weeks from trial treatment start) during the whole observed period, weeks | 1 | number |
| Worst CTCAE grade of pulmonary fibrosis during the whole observed period | 208 | 0, 1, 2, 3, 4, 5 |
| Time when worst CTCAE grade of pulmonary fibrosis was observed (weeks from trial treatment start) during the whole observed period, weeks | 4 | number |
| **RADIOTHERAPY DEFINED BY TRIALS** | | |
| Radiotherapy start date | 1061 | date |
| Radiotherapy end date | 993 | date |
| Radiation dose per fraction, Gy | 239 | number |
| Fraction of radiotherapy | 239 | number |
| Total radiation dose | 1259 | number |
| Discontinuation of radiotherapy | 208 | yes, no |
| Radiotherapy Interruption | 995 | yes, no |
| Days of the radiotherapy interruption | 648 | number |
| Reason for the unscheduled radiotherapy interruption, hematological toxicity | 319 | yes, no |
| Reason for the unscheduled radiotherapy interruption, leucopenia grade 4 | 276 | yes, no |
| Reason for the unscheduled radiotherapy interruption, neutropenia grade 4 | 276 | yes, no |
| Reason for the unscheduled radiotherapy interruption, thrombocytopenia grade 4 | 276 | yes, no |
| Reason for the unscheduled radiotherapy interruption, non-hematological toxicity | 335 | yes, no |
| Reason for the unscheduled radiotherapy interruption, fever grade ≧1 | 319 | yes, no |
| Reason for the unscheduled radiotherapy interruption, esophagitis grade ≧3 | 305 | yes, no |
| Reason for the unscheduled radiotherapy interruption, dermatitis grade ≧3 | 303 | yes, no |
| Reason for the unscheduled radiotherapy interruption, on G-CSF administration | 276 | yes, no |
| Reason for the unscheduled radiotherapy interruption, pneumonitis | 319 | yes, no |
| Reason for the unscheduled radiotherapy interruption, other adverse events | 59 | yes, no |
| Reason for the unscheduled radiotherapy interruption, others except for adverse events | 82 | yes, no |
| Reason for the unscheduled radiotherapy interruption, text data for others | 135 | text |
| Total lung volume exceeding 20 Gy, % | 209 | number |
| Number of portals for the scheduled irradiation | 107 | number |
| Completion of scheduled radiotherapy | 208 | yes, no |
| **CHEMOTHERAPY DEFINED BY TRIALS** | | |
| Chemotherapy start date | 502 | date |
| Chemotherapy end date | 769 | date |
| Discontinuation of chemotherapy | 548 | yes, no |
| Reason for chemotherapy discontinuation, hematological toxicity | 245 | yes, no |
| Reason for chemotherapy discontinuation, non-hematological toxicity | 245 | yes, no |
| Reason for chemotherapy discontinuation, others | 233 | yes, no |
| Chemotherapy interruption | 353 | yes, no |
| Days of the chemotherapy interruption | 58 | number |
| Reason for the unscheduled chemotherapy interruption, hematological toxicity | 50 | yes, no |
| Reason for the unscheduled chemotherapy interruption, non-hematological toxicity | 50 | yes, no |
| Reason for the unscheduled chemotherapy interruption, leucopenia grade 4 | 50 | yes, no |
| Chemotherapy dose reduction | 756 | yes, no |
| Reason for chemotherapy dose reduction, toxicity | 343 | yes, no |
| Reason for chemotherapy dose reduction, others | 343 | yes, no |
| Chemotherapy delay | 661 | yes, no |
| Reason for chemotherapy delay, toxicity | 337 | yes, no |
| Reason for chemotherapy delay, others | 337 | yes, no |
| Completion of scheduled chemotherapy | 102 | yes, no |
| **TUMOR EVALUATION** | | |
| Baseline sum diameter of target lesions, mm | 639 | number |
| Measure meant date of baseline sum diameter of target lesions, date | 637 | date |
| Minimum sum diameter of target lesions during observed period, mm | 627 | number |
| Best reduced rate of target lesion during observed period, % | 627 | number |
| Measurement date of minimum sum diameter of target lesions, date | 625 | date |
| Max growth rate of target lesion during observed period, % | 633 | number |
| Measure meant date of max sum diameter of target lesions, date | 11 | date |
| New lesion emergence during trial treatment | 704 | yes, no |
| New lesion, mediastinal lymph node | 129 | yes, no |
| New lesion, intrathoracic (except for pleural and pericardial lesion) | 129 | yes, no |
| New lesion, pleural and pericardial lesion | 129 | yes, no |
| New lesion, central nerve lesion | 156 | yes, no |
| New lesion, others | 129 | yes, no |
| New lesion, text data about others | 129 | text |
| Diagnosis date of new lesion | 129 | date |
| Diagnosis method of new lesion | 12 | text |
| Best response of target lesion during the whole observed period | 531 | CR, PR, SD, PD, NE |
| Best response of non-target lesion during the whole observed period | 293 | CR, non-CR/non-PD, PD, NE |
| New lesions emergence during the whole observed period | 524 | N, Y |
| Best overall response during the whole observed period | 1249 | CR, PR, SD, PD, NE |
| **TREATMENT COMPLETION AND OUTCOME** | | |
| Completion of trial treatment | 1073 | yes, no |
| Discontinuation of trial treatment | 1073 | yes, no |
| Date of trial treatment discontinuation | 379 | date |
| Discontinuation, progressive disease | 371 | yes, no |
| Discontinuation, adverse event | 371 | yes, no |
| Discontinuation, pneumonitis | 336 | yes, no |
| Discontinuation, lung infection | 336 | yes, no |
| Discontinuation, performance status deterioration | 348 | yes, no |
| Discontinuation, esophagitis | 336 | yes, no |
| Discontinuation, infection except for lung infection | 336 | yes, no |
| Discontinuation, hematotoxicity | 336 | yes, no |
| Discontinuation, other adverse events | 336 | yes, no |
| Discontinuation, text data about other adverse events | 211 | text |
| Discontinuation, patient refusal related to adverse events | 370 | yes, no |
| Discontinuation, patient refusal unrelated to adverse events | 370 | yes, no |
| Discontinuation, death during trial treatment | 370 | yes, no |
| Discontinuation, others | 370 | yes, no |
| Outcome | 1287 | alive, death, unknown |
| Date of last observed survival | 557 | date |
| Date of death | 943 | date |
| Cause of death | 760 | Death by primary disease, Death by other diseases, Death related to treatment, Other, Unknown |
| Cause of death, text data | 407 | text |
| Progressive disease by the end of radiotherapy | 994 | yes, no |
| Date of progression/no progression confirmation by the end of radiotherapy | 985 | date |
| Last day of progression free confirmation by the end of radiotherapy | 948 | date |
| Progressive disease after the end of radiotherapy | 994 | yes, no |
| Date of progression/no progression confirmation after the end of radiotherapy | 950 | date |
| Last day of progression free confirmation after the end of radiotherapy | 195 | date |
| Progressive disease during the whole observed period | 1286 | yes, no |
| Date of progression/no progression confirmation during the whole observed period | 1276 | date |
| Last day of progression free confirmation during the whole observed period | 262 | date |
| Progressed site, within the irradiated area | 802 | yes, no |
| Progressed site, outside of the irradiated area | 802 | yes, no |
| Progressed site, others about an association with the irradiated area | 177 | yes, no |
| Progressed site, primary site | 571 | yes, no |
| Progressed site, any lymph node | 553 | yes, no |
| Progressed site, pulmonary hilum/mediastinum/claviculae | 480 | yes, no |
| Progressed site, other lymph node | 485 | yes, no |
| Progressed site, central nerve | 575 | yes, no |
| Progressed site, bone | 561 | yes, no |
| Progressed site, liver | 559 | yes, no |
| Progressed site, adrenal | 556 | yes, no |
| Progressed site, pulmonary | 527 | yes, no |
| Progressed site, pleural effusion/pleural lesion | 535 | yes, no |
| Progressed site, others | 534 | yes, no |
| Progressed site, others text data | 583 | text |
| **SUBSEQUENT TREATMENT** |  |  |
| Subsequent treatment | 1035 | yes, no |
| Subsequent systemic chemotherapy | 1042 | yes, no, unknown |
| Start date of subsequent systemic chemotherapy no1 | 317 | date |
| Anticancer drug 1-1 | 547 | text |
| Anticancer drug 1-2 | 204 | text |
| Anticancer drug 1-3 | 7 | text |
| Text data about subsequent systemic chemotherapy no1 | 549 | text |
| Start date of subsequent systemic chemotherapy no2 | 174 | date |
| Anticancer drug 2-1 | 299 | text |
| Anticancer drug 2-2 | 70 | text |
| Anticancer drug 2-3 | 8 | text |
| Text data about subsequent systemic chemotherapy no2 | 299 | text |
| Start date of subsequent systemic chemotherapy no3 | 74 | date |
| Anticancer drug 3-1 | 138 | text |
| Anticancer drug 3-2 | 33 | text |
| Anticancer drug 3-3 | 2 | text |
| Text data about subsequent systemic chemotherapy no3 | 138 | text |
| Subsequent radiotherapy | 843 | yes, no, unknown |
| Start date of subsequent radiotherapy no1 | 164 | date |
| Subsequent radiotherapy no1, irradiated cite | 30 | text |
| Subsequent radiotherapy no1, text data of irradiated cite | 166 | text |
| Subsequent radiotherapy no1, irradiated dose per fraction | 34 | number |
| Subsequent radiotherapy no1, irradiated total dose | 49 | number |
| Start date of subsequent radiotherapy no2 | 48 | date |
| Subsequent radiotherapy no2, irradiated cite | 15 | text |
| Subsequent radiotherapy no2, text data of irradiated cite | 50 | text |
| Subsequent radiotherapy no2, irradiated dose per fraction | 13 | number |
| Subsequent radiotherapy no2, irradiated total dose | 18 | number |
| Subsequent surgical procedure | 843 | yes, no, unknown |
| Date of surgical procedure | 48 | date |
| Surgical procedure, text data | 47 | text |

M, male; F, female; COPD, chronic obstructive pulmonary disease; NSCLC, non-small cell lung cancer; EGFR, epidermal growth factor receptor; CTCAE, CTCAE, Common Terminology Criteria for Adverse Events; Gy, gray; CR, complete response; PR, partial response; SD, stable disease; PD, progressive disease; NE, not evaluable

**Table S3. Version of CTCAE of the trials integrated into the database**

| Trials | CTCAE Ver. |
| --- | --- |
| JCOG0301 | 2.0 |
| JCOG9812 | 2.0 |
| NJLCG0601 | 3.0 |
| OLCSG0007 | 2.0 |
| SPECTRA | 4.0 |
| TORG1018 | 4.0 |
| WJTOG0106 | 2.0 |
| WJOG5008L | 3.0 |

CTCAE, Common Terminology Criteria for Adverse Events; JCOG, Japan Clinical Oncology Group; NJLCG, North Japan Lung Cancer Group; OLCSG, Okayama Lung Cancer Study Group; WJOG, West Japan Oncology Group; WJTOG, West Japan Thoracic Oncology Group.
